# Multimodal treatment of perianal fistulas in Crohn’s disease: seton versus anti-TNF versus advancement plasty (PISA): study protocol for a randomized controlled trial

**DOI:** 10.1186/s13063-015-0831-x

**Published:** 2015-08-20

**Authors:** E. Joline de Groof, Christianne J. Buskens, Cyriel Y. Ponsioen, Marcel G. W. Dijkgraaf, Geert R. A. M. D’Haens, Nidhi Srivastava, Gijs J. D. van Acker, Jeroen M. Jansen, Michael F. Gerhards, Gerard Dijkstra, Johan F. M. Lange, Ben J. M. Witteman, Philip M. Kruyt, Apollo Pronk, Sebastiaan A. C. van Tuyl, Alexander Bodelier, Rogier M. P. H. Crolla, Rachel L. West, Wietske W. Vrijland, Esther C. J. Consten, Menno A. Brink, Jurriaan B. Tuynman, Nanne K. H. de Boer, Stephanie O. Breukink, Marieke J. Pierik, Bas Oldenburg, Andrea E. van der Meulen, Bert A. Bonsing, Antonino Spinelli, Silvio Danese, Matteo Sacchi, Janindra Warusavitarne, Ailsa Hart, Nuha A. Yassin, Rory P. Kennelly, Garret J. Cullen, Desmond C. Winter, A. Barney Hawthorne, Jared Torkington, Willem A. Bemelman

**Affiliations:** Department of Surgery, Academic Medical Center, PO Box 22660, 1100, DD Amsterdam, The Netherlands; Department of Gastroenterology and Hepatology, Academic Medical Center, Meibergdreef 9, 1105 AZ Amsterdam, The Netherlands; Clinical Research Unit, Academic Medical Center, Meibergdreef 9, 1105 AZ Amsterdam, The Netherlands; Department of Gastroenterology and Hepatology, Medical Center Haaglanden, Lijnbaan 32, 2512 VA Den Haag, The Netherlands; Department of Surgery, Medical Center Haaglanden, Lijnbaan 32, 2512 VA Den Haag, The Netherlands; Department of Gastroenterology and Hepatology, Onze Lieve Vrouwe Gasthuis, Oosterpark 9, 1091 AC Amsterdam, The Netherlands; Department of Surgery, Onze Lieve Vrouwe Gasthuis, Oosterpark 9, 1091 AC Amsterdam, The Netherlands; Department of Gastroenterology and Hepatology, University Medical Center Groningen, Hanzeplein 1, 9700 RB Groningen, The Netherlands; Department of Surgery, University Medical Center Groningen, Hanzeplein 1, 9700 RB Groningen, The Netherlands; Department of Gastroenterology and Hepatology, Hospital Gelderse Vallei, Willy Brandtlaan 10, 6716 RP Ede, The Netherlands; Department of Surgery, Hospital Gelderse Vallei, Willy Brandtlaan 10, 6716 RP Ede, The Netherlands; Department of Surgery, Diakonessenhuis Utrecht, Bosboomstraat 1, 3582 KE Utrecht, The Netherlands; Department of Gastroenterology and Hepatology, Diakonessenhuis Utrecht, Bosboomstraat 1, 3582 KE Utrecht, The Netherlands; Department of Gastroenterology and Hepatology, Amphia Hospital, Molengracht 21, 4818 CK Breda, The Netherlands; Department of Surgery, Amphia Hospital, Molengracht 21, 4818 CK Breda, The Netherlands; Department of Gastroenterology and Hepatology, St Franciscus Gasthuis, Kleiweg 500, 3045 PM Rotterdam, The Netherlands; Department of Surgery, St Franciscus Gasthuis, Kleiweg 500, 3045 PM Rotterdam, The Netherlands; Department of Surgery, Meander Medical Center, Maatweg 3, 3813 TZ Amersfoort, The Netherlands; Department of Gastroenterology and Hepatology, Meander Medical Center, Maatweg 3, 3813 TZ Amersfoort, The Netherlands; Department of Surgery, VU Medical Center, De Boelelaan 1118, 1081 HZ Amsterdam, The Netherlands; Department of Gastroenterology and Hepatology, VU Medical Center, De Boelelaan 1118, 1081 HZ Amsterdam, The Netherlands; Department of Surgery, Maastricht University Medical Center, P. Debyelaan 25, 6229 HX Maastricht, The Netherlands; Department of Gastroenterology and Hepatology, Maastricht University Medical Center, P. Debyelaan 25, 6229 HX Maastricht, The Netherlands; Department of Gastroenterology and Hepatology, University Medical Center Utrecht, Heidelberglaan 100, 3584 CX Utrecht, The Netherlands; Department of Gastroenterology and Hepatology, Leiden University Medical Center, Albinusdreef 2, 2333 ZA Leiden, The Netherlands; Department of Surgery, Leiden University Medical Center, Albinusdreef 2, 2333 ZA Leiden, The Netherlands; Department of Surgery, Humanitas Hospital, Via Alessandro Manzoni, 56, 20089 Rozzano MI, Milan, Italy; Department of Gastroenterology and Hepatology, Humanitas Hospital, Via Alessandro Manzoni, 56, 20089 Rozzano MI, Milan, Italy; Department of Surgery, St Mark’s Hospital, Watford Road, Harrow, Middlesex HA1 3UJ, London, UK; Department of Gastroenterology and Hepatology, St Mark’s Hospital, Watford Road, Harrow, Middlesex HA1 3UJ, London, England; Department of Surgery, St Vincent’s Healthcare Group, Elm Park, Merrion Rd, Dublin 4, Ireland; Department of Gastroenterology and Hepatology, St Vincent’s Healthcare Group, Elm Park, Merrion Rd, Dublin 4, Ireland; Department of Gastroenterology and Hepatology, Spire Cardiff Hospital, Glamorgan House, Croescadarn Rd, Cardiff, South Glamorgan CF23 8XL, UK; Department of Surgery, Spire Cardiff Hospital, Glamorgan House, Croescadarn Rd, Cardiff, South Glamorgan CF23 8XL, England

**Keywords:** Crohn’s disease, Perianal fistula, Seton, Anti-TNF, Advancement plasty, Quality of life, Cost-effectiveness

## Abstract

**Background:**

Currently there is no guideline for the treatment of patients with Crohn’s disease and high perianal fistulas. Most patients receive anti-TNF medication, but no long-term results of this expensive medication have been described, nor has its efficiency been compared to surgical strategies. With this study, we hope to provide treatment consensus for daily clinical practice with reduction in costs.

**Methods/Design:**

This is a multicentre, randomized controlled trial. Patients with Crohn’s disease who are over 18 years of age, with newly diagnosed or recurrent active high perianal fistulas, with one internal opening and no anti-TNF usage in the past three months will be considered. Patients with proctitis, recto-vaginal fistulas or anal stenosis will be excluded. Prior to randomisation, an MRI and ileocolonoscopy are required. All treatment will start with seton placement and a course of antibiotics. Patients will then be randomised to: (1) chronic seton drainage (with oral 6-mercaptopurine (6MP)) for one year, (2) anti-TNF medication (with 6MP) for one year (seton removal after six weeks) or (3) advancement plasty after eight weeks of seton drainage (under four months anti-TNF and 6MP for one year). The primary outcome parameter is the number of patients needing fistula-related re-intervention(s). Secondary outcomes are the number of patients with closed fistulas (based on an evaluated MRI score) after 18 months, disease activity, quality of life and costs.

**Discussion:**

The PISA trial is a multicentre, randomised controlled trial of patients with Crohn’s disease and high perianal fistulas. With the comparison of three generally accepted treatment strategies, we will be able to comment on the efficiency of the various treatment strategies, with respect to several long-term outcome parameters.

**Trial registration:**

Nederlands Trial Register identifier: NTR4137 (registered on 23 August 2013).

**Electronic supplementary material:**

The online version of this article (doi:10.1186/s13063-015-0831-x) contains supplementary material, which is available to authorized users.

## Background

Crohn’s disease (CD) is a chronic disease that typically affects young adults, with a lifetime risk of fistula development ranging from 14 to 38 % in population-based estimates [1]. Perianal fistulising CD is associated with local pain, discharge and considerable morbidity rates (including sphincter and perineal tissue destruction), resulting in a negative impact on quality of life [2]. The impact on health care resources is enormous due to multiple surgical interventions and costly medication. Yearly, 200 million Euros are spent on anti-tumour necrosis factor inhibitors (anti-TNF) for inflammatory bowel diseases (IBD) in The Netherlands alone, of which 90 % is for CD and 36 million Euros of that is for fistulising CD.

There are several treatment options for complex high fistulas (defined as the involvement of the upper two-thirds of the external sphincter) with one internal opening. Complete closure and fibrosis of the fistula tract can be achieved either via a surgical approach or with medical treatment. Until several decades ago, the most frequently used treatment approach has been surgical seton placement for chronic drainage of the fistula. A seton maintains patency of the tract and eliminates the accumulation of pus, which prevents the recurrent formation of tracts and abscesses, with subsequent low re-intervention rates (10 to 20 %) [3]. However, the seton has been reported to negatively influence quality of life and is associated with a decreased Perianal Disease Activity Index (PDAI) score [4]. Another disadvantage of this technique is that the fistula will not close with the seton *in situ*. It remains unclear when the seton can be removed, and whether the tract heals after removal.

Another surgical treatment option is closure of the internal fistula opening by creating an advancement plasty. This is usually done after primary seton drainage for several weeks. In a recent systematic review, the success rate of endorectal advancement plasty for CD fistulas was 64 %. These results seem quite promising, although almost 10% described faecal incontinence and re-interventions were needed in almost 50 % of patients [5].

With the introduction of anti-TNF agents (infliximab and adalimumab), the treatment for CD fistulas has changed from almost exclusively surgical to placing a much larger emphasis on medical therapy [6]. Since the results of two large trials assessing the benefit of anti-TNF medication, almost all patients receive this medication [7, 8]. The ACCENT I study demonstrated a significant increase in fistula closure with infliximab when compared to placebo treatment (55 % versus 13 % , *P* = 0.001) [7]. In addition, the number of hospitalisations and surgical interventions was significantly reduced by almost 50 % (65 versus 126 procedures per 100 patients, *P* <0.01). An open-label adalimumab trial (CHOICE trial) demonstrated a 39 % fistula healing rate in 88 patients with CD fistulas [8]. Unfortunately, both trials included patients with all fistulising disease (not only perianal), and only a few cohort studies on infliximab present specific data for perianal fistulas, with successful closure rates ranging from 20 to 50 % [9–13]. However, the results are difficult to translate into daily clinical practice since these studies only demonstrated short-term results (follow-up period of 10 to 26 weeks).

There are only a few studies presenting follow-up results over six months. Lichtenstein *et al*. showed that the median length of time during which the fistulas remained closed was three months, with over 50 % re-opening after cessation of medication [14]. The long-term results of the ACCENT II trial showed complete fistula closure in 34 % of patients responding to infliximab therapy after 46 weeks versus 19 % in the placebo group [15]. So far, there are no guidelines for stopping this therapy that is associated with high costs and several side effects. In most studies, more than 60 % of patients experience adverse events (such as headache, infection, and fatigue) [7, 8], and the medication affects the immune system, with potentially serious side effects (such as infections). At present, there is no consensus on treatment for these fistulas, and various approaches are associated with considerable discrepancies in efficiencies and costs.

## Methods/Design

### Study objectives

With this study, we will prospectively assess efficiency as well as efficacy of three generally accepted treatment strategies for high perianal fistulas in patients with CD. With the results, we hope to provide treatment consensus for daily practice with a subsequent reduction in treatment costs.

### Study design

**Fig. 1:**
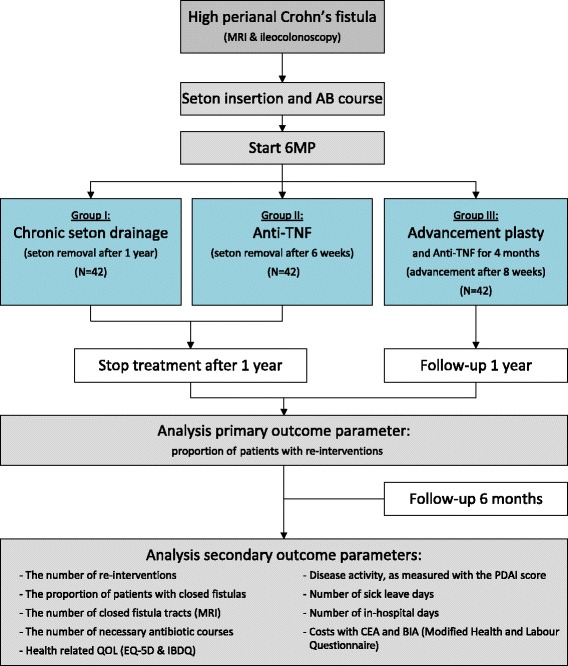
Flowchart of study inclusion and follow-up procedure with outcome parameters. anti-TNF: anti-tumour necrosis factor inhibitors; MRI: magnetic resonance imaging; AB: antibiotic; 6MP: 6-mercaptopurine; N: number; QOL: quality of life; EQ-5D: Euroqol 5D; IBDQ: Inflammatory Bowel Disease Questionnaire; PDAI: Perianal Disease Activity Index; CEA: cost-effectiveness analysis; BIA: budget impact analysis

The PISA trial is a multicentre, randomized controlled trial (RCT). Patients will be randomised to chronic seton drainage, anti-TNF treatment, or advancement plasty (Fig. 1).

### Primary and secondary outcomes

The primary objective of this study is to analyse the number of patients that need a re-intervention due to fistula-related complications (abscesses, recurrent or new tract formation) within one year. The secondary outcomes parameters will be the number of patients with closed fistulas (based on magnetic resonance imaging (MRI) findings) after 18 months, disease activity (based on the Perianal Disease Activity Index (PDAI score)), number of necessary antibiotic courses during fistula treatment and quality of life (measured using the Euroqol 5-dimension questionnaire with 3 levels (EQ-5D-3 L) and the Inflammatory Bowel Disease Questionnaire (IBDQ)). Furthermore, the number of sick leave or in-hospital days, and the related costs will be estimated in a cost-effectiveness analysis (CEA) and a budget impact analysis (BIA).

### Study population

All eligible patients with CD and a newly diagnosed perianal fistula or an existing fistula that has recently become productive or symptomatic will be considered for inclusion. Patients can be included if they meet all of the following inclusion criteria:

1. are ≥18-years-old;
2. have been diagnosed with CD;
3. have at least one high tract (intersphincteric, transsphincteric or suprasphincteric) perianal fistula located in the upper two thirds of the external sphincter;
4. have a fistula with one internal opening, based on MRI imaging (the number of external fistulas does not have to be taken into account);
5. have new fistulas and/or recurrent active fistulas (defined as any producing fistula).

Patients will be excluded if they meet any of the following criteria:

1. Proctitis (defined as any active mucosal inflammation or ulcer >5 mm in the rectum);
2. Anorectal stenosis (defined as the impossibility of introducing a proctoscope);
3. Submucosal fistulas and low intersphincteric fistulas (lower third of external sphincter);
4. Rectovaginal fistula;
5. Multiple internal openings;
6. Seton *in situ* for more than three months;
7. Use of anti-TNF medication during the last three months;
8. Previous anti-TNF medication without any effect on perianal fistulas;
9. Previously demonstrated allergy for anti-TNF medication. If this allergy only concerns the chimeric monoclonal mouse-antibody infliximab, the patient could be randomised for adalimumab;
10. Patients with a stoma;
11. Immunocompromised patients, including those with haematological malignancies, HIV or AIDS, bone marrow transplantation, splenectomy, genetic disorders such as severe combined immunodeficiency, chemotherapy, dialysis, solid organ transplant and long-term immunosuppressant use such as corticosteroids in patients with rheumatoid arthritis);
12. Life expectancy of less than two years;
13. The inability of reading and understanding, and filling in the questionnaires;
14. Dementia or altered mental status that would prohibit the understanding and giving of informed consent.

### Participating centres

Up until now, 13 centres in The Netherlands, including six academic centres, will enrol patients. In addition, a centre in Italy, Ireland and two centres in England will participate.

### Ethics

The study is conducted in accordance with the principles of the Declaration of Helsinki and ‘good clinical practice’ guidelines. The protocol has been approved by the Medical Ethical Committee of the Academic Medical Center in Amsterdam (METC 2013_201). Consent was also obtained from the participating centres (Additional file 1). Patients with CD and presenting with a perianal fistula will be counselled, and written informed consent will be obtained from all patients if the inclusion and exclusion criteria are met.

### Study outline

Eligible patients will be recruited at the IBD outpatient department of each participating medical centre. At inclusion, an MRI will be performed to assess the course of the fistula tracts, the number of internal openings and to exclude concurrent perianal abscesses. Prior to randomisation, an ileocolonoscopy is necessary to exclude proctitis. In case it is not possible to perform an ileocolonoscopy, a sigmoidoscopy and Magnetic Resonance Enteroclysis (MRE) must be performed prior to randomisation. If during this investigation luminal disease activity is observed, patients will receive standard treatment, starting with an eight-week prednisolone course, dosing at the discretion of the treating physician, together with an immunomodulator of either oral 6-mercaptopurine (6MP) 1 to 1.5 mg/kg or oral azathioprine 2 to 2.5 mg/kg or subcutaneous methotrexate 25 mg/week. At week 12, step-up to anti-TNF (infliximab or adalimumab) in case of insufficient response of luminal disease activity to steroids or the immunomodulator is allowed. These patients will not be excluded from the trial.

### Randomisation and blinding

Random block randomisation of consented study participants will be performed by an internet randomisation module prepared by the Clinical Research Unit of the Academic Medical Center. Randomisation will not be stratified. Due to the comparison of surgical and medical treatment strategies in this study, blinding to the treatment allocation for patient and medical staff is not possible. The statistician will analyse the data blinded for treatment allocation.

### Chronic seton drainage

Treatment for the seton group is scheduled to last for one year. Seton (vessel loop) placement will be performed under general anaesthesia in a day care setting and patients will receive a two-week antibiotic course of ciproflaxin 500 mg twice a day; in case of non-responsiveness, patients will switch to metronidazole 500 mg three times a day for two weeks). Medical treatment with 6MP (Puri-nethol by Aspen Pharma Trading Limited, Dublin, Ireland) will be added 1 to 1.5 mg/kg.

### Anti-TNF

Patients randomly allocated to the second treatment arm will also start with the insertion of a seton and a two-week antibiotic course. Then, anti-TNF treatment will be initiated combined with 6MP. The anti-TNF choice (infliximab (REMICADE by Janssen Biologics Inc., Pennsylvania, USA) and adalimumab (HUMIRA by AbbVie Biotechnology GmbH, Wiesbaden Germany)) will be left to the discretion of the treating gastroenterologist. The anti-TNF agent consists of infliximab 5 mg/kg at the beginning of treatment, which will be repeated at two and six weeks as a loading dose. After this, treatment will be scheduled for every eight weeks. If a patient is non-responsive to the treatment dose escalation to 5 mg/kg every six weeks is permitted. When adalimumab is the preferable anti-TNF agent, a loading dose of 160 mg at the start of treatment is required. After two weeks, the dosage will be reduced to 80 mg, and then continued with 40 mg every two weeks. If a patient is non-responsive, dose escalation is allowed to up to 40 mg a week. After six weeks, the seton will be removed. Anti-TNF treatment will be continued for up to one year.

### Advancement plasty

In the advancement plasty group, treatment also starts with the insertion of a seton and a two-week antibiotic course. Subsequently this group will start with anti-TNF treatment combined with 6MP. The advancement plasty will be performed in a day-care setting (directly following seton removal) within eight to 10 weeks after randomisation, when the anti-TNF agent has reached therapeutic levels. The anti-TNF medication will be continued until four months after randomisation. For optimal results the procedure must be performed by a specialised colorectal surgeon. When participating centres lack a qualified surgeon, the patient will be referred to the Academic Medical Center.

### Statistical analysis

The outcome parameters will be analysed with appropriate statistical tests by a statistician on an intention-to-treat basis, using the statistical program IBM SPSS Statistics for Windows, Version 21.0. Armonk, NY: IBM Corp.. A two-tailed *P* <0.05 is considered statistically significant. Analyses will be presented with 95 % confidence intervals. The primary and secondary outcome parameters, the proportion of patients in need of re-interventions and the proportion of patients with closed fistulas respectively, will be compared with chi-squared testing. The difference in number of re-interventions between the three groups will be compared using Kruskal Wallis testing. Additional mixed-models repeated measures analysis of variance will be used to investigate whether there is a different pattern of change over time between the three study arms in the four IBDQ dimensions and the EQ 5D-3 L.

### Sample size calculation

The principal analysis will consist of an intention-to-treat comparison of the proportions of patients with fistula-related interventions in the three treatment groups. The goal is to test superiority of chronic seton drainage over the other two groups. Based on the available literature with re-interventions in 50 % of patients in the anti-TNF group and advancement plasty group, an absolute reduction to between 30 and 20 % of patients needing a re-intervention in the chronic seton group is considered clinically relevant and feasible. The sample size needed to detect this difference with two-sided chi-squared testing equals 42 patients per group, or 126 patients overall (alpha 0.05, power 80 % and 5 % dropout rate).

### Data collection and monitoring

Patients will be seen at the outpatient clinic at regular intervals after randomisation by the surgeon or the gastroenterologist, depending on the assigned treatment strategy. Thereafter, all patients will be seen at six, 12 and 18 months after inclusion. Other visits will be scheduled on indication. During these contacts the PDAI score will be assessed. Patients will fill in health-related quality of life questionnaires (EQ-5D-3 L, IBDQ and the Modified Health and Labour Questionnaire) at inclusion and every three months after. The questionnaires will be completed by patients electronically using LimeSurvey 1.90 via https://www.limesurvey.org/en/, a personal access code sent by email. Patients not willing or unable to complete the online questionnaires will receive identical paper questionnaires at their home address, accompanied by a free return envelope. An electronic case report form will include general patient data. Patients will be followed by a trial nurse to assess complications, re-admissions, duration of hospital stay and the number of sick leave days. After 18 months patients will undergo an MRI to assess fistula closure, or at the suspicion of an abscess or new fistula tract. An evaluated MRI-based score will be used to classify disease severity for perianal fistulising CD [16].

### Patient safety

All three treatment strategies are generally accepted, therefore there is no additional risk. However, an interim review will be performed after one year of follow-up for one third of included patients (n = 42), and after one-year of follow-up for two thirds of included patients (n = 84). The Data and Safety Monitoring Board (DSMB) will be supplied with the number of (serious) adverse events and other outcome parameters in all three groups at this time. If there is a skewed distribution of the number of (serious) adverse events between the groups, an efficacy analysis can be performed at the discretion of the DSMB. Following this interim analysis, the DSMB will advise the study Steering Committee upon continuation of the trial.

### Cost-effectiveness and cost-utility

An economic evaluation will be performed from a societal perspective as a cost-effectiveness and cost-utility analysis with a time horizon of 18 months. The primary economic outcomes are the costs per patient with re-interventions and the costs per quality adjusted life year (QALY), respectively. The CEA closely relates to the primary clinical outcome measure; the cost-utility analysis is performed to support health policymakers in allocating health care resources across patient groups, health care settings and interventions. Incremental cost-effectiveness ratios of each alternative treatment (anti-TNF or advancement plasty) against chronic seton drainage as the reference strategy will be calculated as the extra costs per additional patient without re-intervention(s) and the extra costs per additional QALY. To account for sampling variability, differences between groups will be assessed by calculating the 95 % confidence intervals, after correction for bias, and using accelerated non-parametric bootstrapping. Results will be displayed graphically with cost-effectiveness planes and cost-effectiveness acceptability curves for willingness-to-pay values up to €100,000. Sensitivity analyses will be performed for the unit costs of (chronic) seton drainage, advancement plasty and anti-TNF medication. Sensitivity analysis will also be performed for different (Dutch and UK general population based) health utility scoring algorithms used to derive QALYs. Considering the time horizon of 18 months, we will discount the effects and costs during the second year of follow-up.

Data on health care resource use (index interventions, re-interventions, hospitalisations and out-of-hospital care), health-related out-of-pocket expenses by patients and productivity losses resulting from sick leave will either be retrieved from hospital information systems, or gathered with clinical report forms and tailored patient questionnaires. Unit costing of resources used will be in accordance with current national guidelines (CvZ/EUR-iMTA: College voor Zorgverzekeringen/Erasmus Universitu Rotterdam-institute for Medical Technology Assessment). In case of productivity losses resulting from sick leave or lower efficiency while at work, the friction cost approach to costing will be applied. All costs will be expressed in Euros for the base year 2013. Costs borne in other calendar years will be price indexed.

Perianal fistulas in CD may heavily affect a person’s quality of life. Affected patients experience pain and feel restricted in daily activities as well as in sexual activities. At present, the PDAI is the gold standard for evaluating the severity of perianal disease. It includes five items: discharge, pain, restriction of sexual activity, type of perianal disease and degree of induration. Each category is graded on a five-point Likert scale ranging from no symptoms to severe symptoms. The PDAI will be disseminated at baseline and half-yearly thereafter. In addition, the EQ-5D-3 L will be used to gather health status profiles over time (baseline and at quarterly intervals). These profiles will be evaluated by applying existing time trade-off based health utility scoring algorithms from previous research among general adult populations in The Netherlands and the UK [17, 18]. QALYs will then be calculated by taking the product sum of the resulting health utilities and the length of the periods in-between successive measurements. All patient outcome data will be analysed as repeated measurements by linear mixed modelling.

### Budget impact

The short- and mid-term budget impacts of the three treatment strategies will be assessed from governmental and insurer perspectives in accordance with a recent International Society for Pharmacoeconomics and Outcomes Research (ISPOR) guideline [19]. Budget impact analyses may guide reimbursement decisions and may influence price and volume negotiations between insurer and health care provider. In this study, the budget impact analyses will be incidence-based, concerning patients with CD that are newly diagnosed with a perianal fistula. At the same time, the analyses will be patient-based, covering all health care costs observed during the 18 months of follow-up. At this time, the treatment strategies will presumably end up with comparable proportions of patients with complete fistula closure.

The governmental perspective is chosen to help set priorities in health care optimization while simultaneously considering the wider implications of the seton drainage, anti-TNF medication and advancement plasty beyond the health care sector (societal impact). The governmental perspective further includes an impact assessment on budgets for institutions for specialist medical care, self-employed medical specialists and (expensive) drugs (Rijksbegroting 2012, premie gefinancierde zorg (http://www.rijksbegroting.nl/2012/voorbereiding/begroting,kst160371_3.html). The insurer perspective is chosen to assess the net financial consequences of replacing anti-TNF drug therapy with the seton drainage or advancement plasty strategies. Hence, against the base case scenario of predominantly anti-TNF medication supply, we will assess the impact of an instant, gradual or partial shift to seton drainage or to advancement plasty. As with the CEA, sensitivity analyses will be performed for the unit costs of (chronic) seton drainage, advancement plasty and anti-TNF medication. The time horizon for all budget impact assessments will be four years and will be reported for each successive calendar year.

## Discussion

High perianal fistulas in patients with CD have an enormous impact on patients’ quality of life, as well as on health care systems [2]. Our goal is to provide evidence for optimal treatment resulting in (inter)national consensus of all disciplines involved in the treatment of these patients. All three treatment approaches are currently included in the basic health insurance packages and the Dutch Exceptional Medical Expenses Act 1968 (AWBZ). In the three study groups, an optimal combination of available treatment regimens will be provided. Every patient starts with seton drainage to create a patent tract and to reduce inflammation, preventing the accumulation of pus. This will be accompanied by the prescription of a two-week antibiotics course, since the ADAFI study demonstrated that anti-TNF therapy is more effective in combination with a course of ciprofloxacin [20]. The anti-TNF medication will be combined with 6MP to optimise the result of the anti-TNF medication, since Colombel *et al*. established that patients receiving combination therapy were more likely to have corticosteroid-free clinical remission [21]. In the ECCO guideline of 2010, treatment with azathioprine or 6MP is recommended in combination with appropriate surgical therapy for complex perianal CD, in spite of a lack of clinical trials. Therefore, the patients in the surgical arms will also be treated with azathioprine or 6MP [22]. Since it has been described that the success rate of advancement plasty is slightly higher when combined with anti-TNF therapy (50 % versus 65 %) [5], this group will receive medication for four months. Once the fistula is closed, anti-TNF medication is not likely to have any additional value and will be discontinued.

Based on the available literature, superiority for chronic seton drainage with respect to the number of re-interventions is expected. Both anti-TNF medication and advancement plasty have been described to have up to a 50 % re-intervention rate due to recurrent fistulas or abscess formation, whereas this is hardly seen in chronic seton drainage due to preservation of a patent tract. We hypothesise that after 18 months, no differences will be found in the number of patients with draining fistulas for all three groups described previously. Studies presenting the long-term results for seton drainage describe up to 40 % closure of fistulas after seton removal [23–27]. This is in line with the sparse literature on long-term results for anti-TNF medication or advancement plasty results. For advancement plasty, the initial results show up to 65 % success rates, but recurrences occur in a substantial subset of patients, and this technique has been associated with decreased functional outcome with faecal incontinence occurring in 10 % of cases [5]. For anti-TNF medication, a 40 to 60 % initial closure rate has been described, but one study with long-term results demonstrated that 50 % of the responding patients had recurrent fistulas after cessation of therapy [14]. When combining these results, it can be expected that surgical drainage will be highly cost-effective. Treatment strategies will be stopped after one year, while patients will be followed up on for another six months, in order to comment on long-term results. With the comparison of these three groups, we will be able to comment on the efficiency of the various treatment strategies with respect to several long-term outcome parameters.

## Trial status

In total, we currently included 16 patients in the PISA trial at the time of submission of the protocol to *Trials* (17 June 2015). The first patient was included in the Academic Medical Center on 1 November 2013. In 2014 and 2015, the other participating centres were added to the trial. In the supplementary file, dates of consent in the participating centres are specified.

## Additional file

Additional file 1:
**Dates of consent of participating centres.**

## Abbreviations

6MP: 6-mercaptopurine
anti-TNF: anti-tumour necrosis factor
BIA: budget impact analysis
CD: Crohn’s disease
CEA: cost-effectiveness analysis
DSMB: Data and Safety Monitoring Board
EQ-5D-3 L: Euroqol 5-dimension 3 levels
IBD: inflammatory bowel diseases
IBDQ: Inflammatory Bowel Disease Questionnaire
MRI: Magnetic Resonance Imaging
PDAI: Perianal Disease Activity Index
PISA: Multimodal treatment of Perianal fistulas in Crohn’s disease: Seton versus anti-TNF versus Advancement plasty
RCT: randomised controlled trial
