# Supplementary material for: Multimodal treatment of perianal fistulas in Crohn’s disease: seton versus anti-TNF versus advancement plasty (PISA): study protocol for a randomized controlled trial
Source: Trials. 2015 Aug 20;16:366. doi: 10.1186/s13063-015-0831-x (PMC4545975; doi:10.1186/s13063-015-0831-x)
Supplement: Additional file 1: — Dates of consent of participating centres. [file 13063_2015_831_MOESM1_ESM.pdf]

Supplementary file – dates of consent in participating centres

Participating centres with consent:

1. Academic Medical Center, Amsterdam, The Netherlands (21-08-2013)
2. Medical Center Haaglanden, Den Haag, The Netherlands (24-02-2014)
3. Onze Lieve Vrouwe Gasthuis, Amsterdam, The Netherlands (31-03-2014)
4. University Medical Center Groningen, Groningen, The Netherlands (31-03-2014)
5. Hospital Gelderse Vallei, Ede, The Netherlands (16-05-2014)
6. Diakonessenhuis Utrecht, Utrecht, The Netherlands (20-06-2014)
7. Amphia Hospital, Breda, The Netherlands (16-05-2014)
8. St Franciscus Gasthuis, Rotterdam, The Netherlands (09-09-2014)
9. Meander Medical Center, Amersfoort, The Netherlands (20-06-2014)
10. VU Medical Center, Amsterdam, The Netherlands (22-04-2014)
11. Maastricht University Medical Center, Maastricht, The Netherlands (20-06-2014)
12. University Medical Center Utrecht, Utrecht, The Netherlands (12-09-2014)
13. Leiden University Medical Center, Leiden, The Netherlands (18-07-2014)
14. Humanitas Hospital, Rozzano, Milan, Italy (13-01-2015)

Centres currently obtaining consent:

15. St Mark's Hospital, London, England
16. St Vincent's Healthcare Group, Dublin, Ireland
17. Spire Cardiff Hospital, Cardiff, England
